# Manifold Approximating Graph Interpolation of Cardiac Local Activation Time

**DOI:** 10.1109/TBME.2022.3166447

**Published:** 2022-09-19

**Authors:** Jennifer Hellar, Romain Cosentino, Mathews M. John, Allison Post, Skylar Buchan, Mehdi Razavi, Behnaam Aazhang

**Affiliations:** Electrical and Computer Engineering Department, Rice University, Houston, TX 77005 USA.; Electrical and Computer Engineering Department, Rice University, USA.; Electrophysiology Clinical Research and Innovations Department, Texas Heart Institute, USA.; Electrophysiology Clinical Research and Innovations Department, Texas Heart Institute, USA.; Electrophysiology Clinical Research and Innovations Department, Texas Heart Institute, USA.; Department of Cardiology, Texas Heart Institute, USA.; Electrical and Computer Engineering Department, Rice University, USA.

**Keywords:** Ablation, local activation time, graph signal processing, semi-supervised learning

## Abstract

**Objective:**

Local activation time (LAT) mapping of cardiac chambers is vital for targeted treatment of cardiac arrhythmias in catheter ablation procedures. Current methods require too many LAT observations for an accurate interpolation of the necessarily sparse LAT signal extracted from intracardiac electrograms (EGMs). Additionally, conventional performance metrics for LAT interpolation algorithms do not accurately measure the quality of interpolated maps. We propose, first, a novel method for spatial interpolation of the LAT signal which requires relatively few observations; second, a realistic sub-sampling protocol for LAT interpolation testing; and third, a new color-based metric for evaluation of interpolation quality that quantifies perceived differences in LAT maps.

**Methods:**

We utilize a graph signal processing framework to reformulate the irregular spatial interpolation problem into a semi-supervised learning problem on the manifold with a closed-form solution. The metric proposed uses a color difference equation and color theory to quantify visual differences in generated LAT maps.

**Results:**

We evaluate our approach on a dataset consisting of seven LAT maps from four patients obtained by the CARTO electroanatomic mapping system during premature ventricular complex (PVC) ablation procedures. Random sub-sampling and re-interpolation of the LAT observations show excellent accuracy for relatively few observations, achieving on average 6% lower error than state-of-the-art techniques for only 100 observations.

**Conclusion:**

Our study suggests that graph signal processing methods can improve LAT mapping for cardiac ablation procedures.

**Significance:**

The proposed method can reduce patient time in surgery by decreasing the number of LAT observations needed for an accurate LAT map.

## I. Introduction

**Fig. 1.**
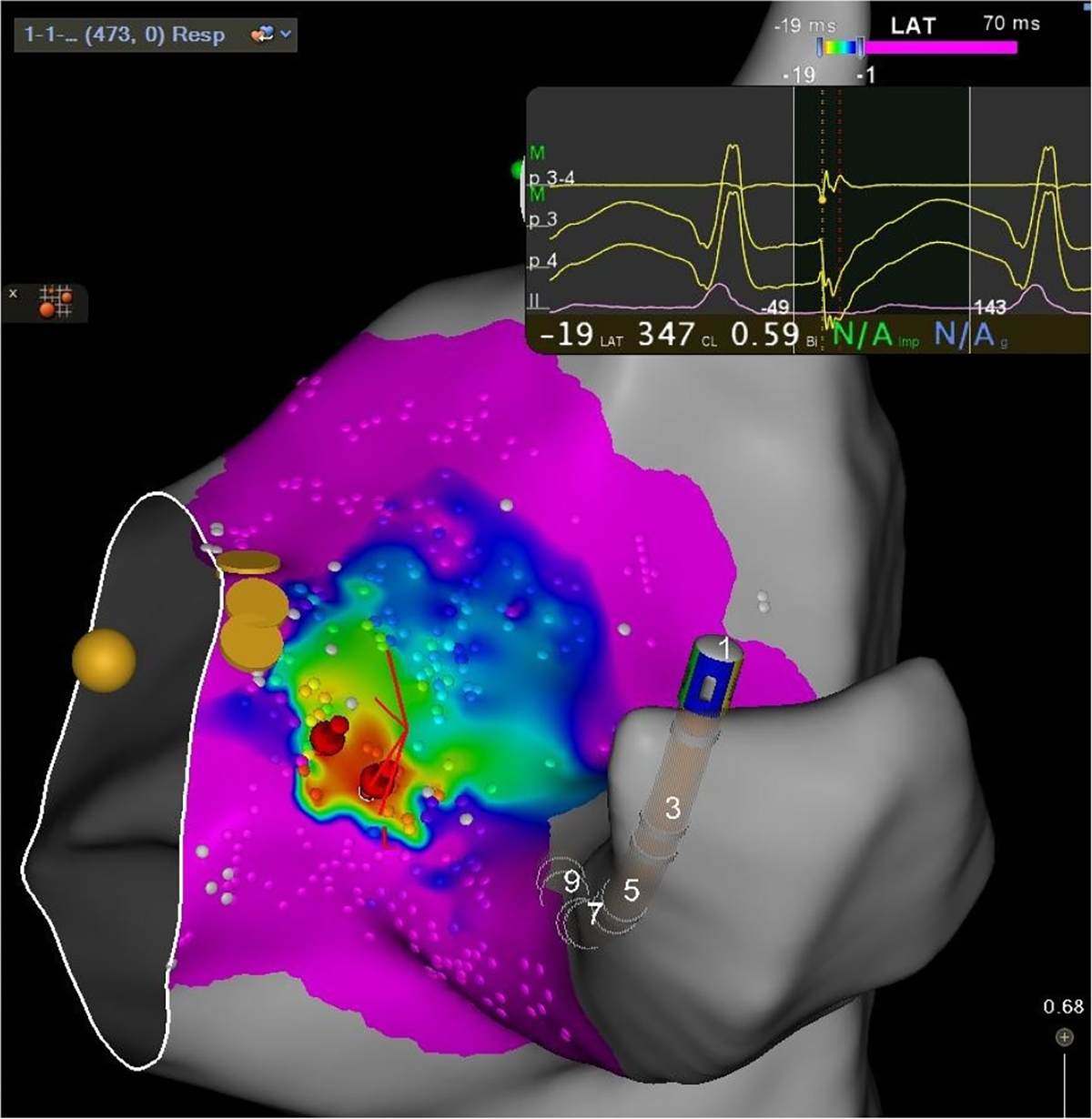
A typical LAT map (3D rendering of the LAT signal) where small dots represent LAT sample points and color encodes the LAT value, with red being earliest activation and purple being latest.

Local activation time (LAT) maps visually characterize the electrical activation propagation which triggers cardiac tissue depolarization and muscle contractions to pump blood throughout the body. A detailed LAT map as in Fig. 1 allows cardiac surgeons to accurately diagnose and treat cardiac arrhythmias with targeted ablation of tissues exhibiting abnormal activation [1]. Between 2000 and 2013, more than 500,000 such ablation procedures were performed [2]. Recent work in automated analysis of LAT maps shows promising results that could improve clinical outcomes even beyond what is possible now [3], [4]. The benefits of these developments, however, requires accurate LAT maps which are currently slow and difficult to obtain.

During an ablation procedure, the LAT map is generated prior to and after each ablation to analyze the original arrhythmia circuit and effect of each intervention. LAT samples are extracted from local unipolar or bipolar electrogram (EGM) signals recorded from the cardiac surface at multiple locations using an electroanatomic mapping (EAM) system [5], [6]. For a given EGM recording, the cycle window corresponding to one beat is selected, and a particular signal feature e.g. the most negative slope is identified and annotated. The difference in time between that feature (*t*_*i*_) and the same feature appearance at a reference location EGM (*t*_0_) is the local activation time *LAT*_*i*_ = *t*_*i*_ − *t*_0_. Despite improved catheter designs that allow simultaneous multi-point recording, the overall LAT mapping process remains slow, taking several minutes to obtain sufficient observations to interpolate and construct a useful map.

One proposed solution to this problem hinges on the fact that the most important LAT map feature for ablation treatment is the point of earliest activation. In [7], the authors propose an automated algorithm to identify ideal LAT recording locations to minimize error in predicting specifically the site of earliest activation. This approach, however, devalues the benefits of diagnosis and treatment based on a complete and accurate LAT map of the whole circuit. Significant work has also been done to develop automated LAT annotation methods [6], [8], [9] to replace manual EGM annotations, which would speed up the process some but not reduce the overall number of observations required. Methods for estimating LAT maps and performing diagnosis from non-invasive electrocardiographic imaging vests have been proposed [4], [10] but with prohibitively low accuracy for clinical use [11].

A more general and direct solution to reduce LAT mapping time is to improve the interpolation of the LAT observations such that an accurate map can be generated with fewer observations. The need for re-examination of interpolation methods for LAT mapping has already been noted in the literature [12], [13], but presently only a few groups have attempted to address it. Current interpolation methods include cubic spline interpolation [14], radial basis functions [15], Gaussian processes [16], [17], physics-informed neural networks [18], [19], and graph convolutional neural networks [20]. Of these, [14] and [15] do not interpolate on the manifold surface, while [16] and [17] assume a smooth Gaussian prior on a transformation of the data.

A few works seek to incorporate physical equations governing activation propagation with promising initial results on simulated patient data [18], [19]. These, however, as well as [20], rely on neural network solutions that are fairly computationally intensive and require large amounts of training data, making clinical implementation potentially more challenging. The authors therefore incorporate mostly synthetic data for training and validate on only very small clinical datasets. In addition, neural networks generally provide very little insight about the solution learned during training, and their interpretation and theoretical guarantees are still an active area of research.

Two recent works [21], [22] also integrate an underlying physical model to address the closely related problem of estimating conduction velocity and tissue fiber direction, but they primarily use high-density LAT maps as an input, therefore it remains unclear how applicable these approaches would be to the LAT interpolation problem.

LAT interpolation remains an open and challenging research problem for a number of reasons. The patient-specific nature of the data means that each patient has both a unique cardiac anatomical structure and a distinct electrical activation pattern that must be taken into account to obtain an accurate LAT map. The underlying structure, the surface of the heart, is an irregular domain that is hard to represent mathematically and leverage for interpolation. And the LAT signal itself is sparse and irregularly sampled with respect to the surface. Our task is therefore to interpolate a sparse and irregular signal on an irregular domain where both the signal and domain are patient-specific.

To address these issues, we use graph signal processing to perform LAT interpolation on the surface of the heart, formally represented as a manifold. Graph signal processing allows us to apply the traditional signal processing toolbox to an irregular domain, namely, the surface of the heart. Similar graph frameworks utilizing the underlying manifold governing the data have been successfully used in a variety of other applications [23]–[28]. We test and validate our method on a clinical patient dataset of seven LAT maps from four patients undergoing ablations for premature ventricular contractions (PVCs), a type of cardiac arrhythmia. This method, Manifold Approximating Graph Interpolation of Cardiac LAT (MAGIC-LAT), follows directly from an intuitive re-formulation of the interpolation problem into a semi-supervised learning framework which incorporates key results in the fields of graph signal processing [29], [30] and geometric manifold learning [31]–[35]. A preliminary version of this work has been reported [36].

We will show that our method has an insightful interpretation as a graph filter with respect to the manifold surface. This interpretation, coupled with the knowledge that activation is governed by the physics of electrical depolarization waves in tissue, suggests that extensions of our framework to incorporate new filter-based signal processing methods may generate interesting insight into the complex patterns of electrical activation propagation.

We also address the problem of properly sub-sampling the recorded LAT signal to perform a re-interpolation that is close to the real clinical scenario. To the best of our knowledge, we propose the first non-uniform sampling distribution designed to mimic the typical distribution of LAT observations obtained during ablation procedures. In particular, our distribution results in a sub-sampled signal with a high concentration of mid- to early-activation points, since mapping technicians more densely sample those regions as they search for the point of earliest activation.

Finally, while investigating and comparing the accuracy of various LAT interpolations, we found that conventional metrics (mean squared error, mean absolute error, etc.) do not intuitively and correctly quantify the true *perceived* accuracy of a given LAT map. We therefore propose and implement a new metric for this application, named Mean Delta-E (MDE) and inspired by the powerful color distance measure ΔE* [37]. MDE leverages the fact that LAT values are represented as colors on the LAT map and directly quantifies the perceptual difference in estimated versus true LAT values by measuring the corresponding color difference in the uniform Commission Internationale de l’Éclairage L*a*b* (CIELAB) colorspace.

The paper is organized as follows. Section II describes the dataset used for testing and analysis of this method. Section III outlines our methods and contributions: Section III-A details our data pre-processing protocols, Sections III-B and III-C describe the mathematical framework of our graph-based approach, Section III-D details our perception-based performance metric, and Section III-E defines our sub-sampling protocol. Section IV describes key aspects of our experimental setup. Section V evaluates the performance of our method on the patient dataset. Sections VI and VII discuss the results and the filtering interpretation.

## II. Dataset

Our dataset consists of seven LAT maps from four patients obtained by the CARTO electroanatomic mapping system during PVC ablation procedures. All data was collected retrospectively from patients that underwent PVC ablation under a protocol approved by the IRB at Baylor St. Luke’s Medical Center (BSLMC). A summary of the dataset is provided in Table I. Of the seven maps, four characterize patients exhibiting a PVC arrhythmia, and the remaining three sinus rhythm. Each map is comprised of

1. A triangular mesh approximating the anatomy of the cardiac chamber, provided in Biosense Webster Triangulated Mesh file format. The triangular mesh {𝒱,ℱ} approximates the geometry of the surface with
  - 𝒱, an ordered set of vertices $v_{i}\in {\mathbb{R}}^{3}$ for *i* = 1, 2, . . . , *n* which are locations on the cardiac surface, and
  - ℱ⊂𝒱×𝒱×𝒱, a set of triangular faces where a face $\left(v_{i},v_{j},v_{k}\right)\in ℱ$ defines edges between the three vertices *v*_*i*_, *v*_*j*_, and $v_{k}\in 𝒱$.
2. A set of LAT sample values *s*_1_, . . . , *s*_*m*_ and corresponding locations in ${\mathbb{R}}^{3}$. Note that the sample locations do not always coincide with a vertex of the mesh.

In addition, we validate our method on the left atrium case simulated in [18], in which a monodomain model for tissue and the Fenton Karma model for cells was used with homogeneous conductivity at 0.1 mm^2^/ms in the entire domain. We refer to this simulated case as “Map S”.

## III. Methods

Let ℳ be the smooth manifold that is the surface of the heart. In general, we want an interpolant function f:ℳ→ℝ which maps any location on the manifold to its LAT value such that the function generalizes to the whole manifold. In our dataset, the triangular mesh {𝒱,ℱ} approximates the manifold ℳ so we instead pursue a discrete interpolant function f:𝒱→ℝ which outputs the LAT value for any vertex $v_{i}\in 𝒱$.

### A. Pre-Processing

Due to measurement imprecision, LAT sample coordinates do not exactly coincide with vertex coordinates in 𝒱. To process the signal on the surface, we assign each LAT value *s*_1_, . . . , *s*_*m*_ to the nearest surface vertex in 𝒱. Moreover, during the ablation procedure, the mapping technician manually selects and discards LAT observations that are inconsistent with nearby observations, due to measurement noise or annotation error. To replicate this process, we visually inspect the LAT observations and remove locally anomalous samples prior to any computation. In the case of LAT Map 6, which contains a prohibitively large number of observations for manual examination (see Table I), we automate the process and remove any LAT observation which differs from the average of its 5 nearest neighbors within 5 mm by more than 30 ms. This gives us a subset of sampled vertices ${𝒱}_{𝒮}$.

### B. Graph Construction

We first transform the mesh into an undirected graph 𝒢={𝒱,ℰ,W} such that the set of vertices 𝒱 with |𝒱|=n is unchanged. An edge (*v*_*i*_, *v*_*j*_) is included in the set of edges ℰ if and only if *v*_*i*_ and *v*_*j*_ form two corners of a face in ℱ.

Note that this graph 𝒢 is purely mesh-based and does not contain any information about the possible causes for high-frequency LAT signal variation (scar tissue blocking electrical activation, opposite edges of a re-entrant cycle meeting, etc.). Therefore, we update 𝒢 and remove functional connections (edges) that are not physiologically present for one of those reasons. To do this, we compute a quantization of the manifold using Nearest Neighbors (NN) applied onto the input LAT observations. From this quantization, we are able to remove any graph edges $\left(v_{i},\;v_{j}\right)\in ℰ$ with a Δ*LAT*_*ij*_ ≥ 50 ms, resulting in a sparsified graph that is a more descriptive representation of the physiological manifold.

Our interpolant function f:𝒱→ℝ is a graph signal defined on the vertices of the graph and represented as a vector $f\in {\mathbb{R}}^{n}$ where the *ith* component of vector **f** represents the function value at the *ith* vertex in 𝒱. Our partially sampled LAT graph signal $f_{s}\in {\mathbb{R}}^{n}$ then has non-zero values only for vertices in the sampled set,

$$f_{s}\left(v_{i}\right)=\{\begin{array}{ll} s_{i} & v_{i}\in {𝒱}_{𝒮}\\ 0 & \text{otherwise.} \end{array}$$

The graph Laplacian **L** forms a real symmetric matrix that encapsulates the connectivity of 𝒢 [30]. To construct it, we consider the desired properties of a discrete Laplacian operator, including symmetry, locality, positive weights, and convergence [38]. For triangular mesh operations in computer graphics, the ubiquitous cotangent weights and associated Laplacian fulfill most requirements, with the exception of positive edge weights [39]. We therefore use the recent implementation of a robust contangent-based Laplacian with guaranteed positive edge weights for our application [40].

### C. Re-Formulation of the Interpolation Problem

Our graph construction defines a sampling of the manifold ℳ at the graph vertices 𝒱, with a corresponding discrete spatial sampling **f** = [*s*_1_, . . . , *s*_*n*_] of the underlying continuous LAT signal. Since we do not have a complete signal **f**, we want to estimate it with f*, a smooth interpolation of the partially sampled signal **f**_**s**_. To do this, we solve the following optimization problem,

(1) $$f^{*}=\arg\underset{f\in {\mathbb{R}}^{n}}{\min}{\left\|M_{l}\left(f-f_{s}\right)\right\|}_{2}^{2}+\alpha {\left\|M_{u}f\right\|}_{2}^{2}+\beta f^{T}Lf.$$

Here, the first term is signal assignment loss with **M**_**l**_ a diagonal binary matrix corresponding to labelled (known) samples *s*_1_, . . . , *s*_*m*_. The second term is a standard Tikhonov regularization, controlling the sum of magnitudes squared of the unknown samples selected by the diagonal binary matrix **M**_**u**_. Note that **M**_**l**_ + **M**_**u**_ = **I**, the *n* × *n* identity matrix. The third term, a Dirichlet regularization, controls signal smoothness; it is the weighted sum of adjacent signal differences squared,

$$f^{T}Lf=\frac{1}{2}\sum_{i,j=1}^{n}w_{ij}{\left(f_{i}-f_{j}\right)}^{2}.$$

This manifold regularization approach has been developed and studied in graph signal processing literature for some time (see [31], [32], [35]), but not in the context of this application. The solution to (1), for which the proof can be found in our Supplementary Document, is

(2) $$f^{*}={\left(M_{l}+\alpha M_{u}+\beta L\right)}^{-1}f_{s}.$$

For our application, optimal regularization coefficients *α* = 10^−5^ and *β* = 10^−2^ were obtained during cross-validation over values

$$\alpha =\left[10^{-5},10^{-4},10^{-3},10^{-2},10^{-1},1,10\right],$$

$$\beta =\left[10^{-5},10^{-4},10^{-3},10^{-2},10^{-1},1,10\right].$$

In particular, further increasing *β*, the coefficient of smoothing, did not significantly change the interpolation performance, but reducing it resulted in consistently higher error. Intuitively, we expect a certain level of smoothness in the signal which this parameter captures.

Interestingly, the expression in (2) which gives us our semi-supervised learning solution may also be viewed from a filtering perspective which we discuss in Section VI. The solution in (2) is hereafter referred to as MAGIC-LAT.

### D. Performance Metric

The key factor in choosing or designing a method evaluation metric is the target application. In this case, we know that a cardiac surgeon visually inspects the LAT map and identifies features based on relative color differences. Therefore, we want a metric that will accurately quantify the perceived color differences for generated maps versus the ground truth.

**Fig. 2.**
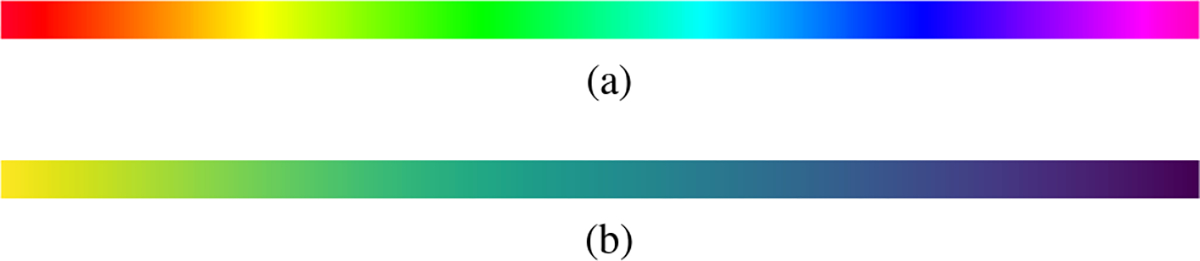
(a) The *gist_rainbow* colormap is non-sequential and non-uniform. (b) The *viridis* colormap is sequential and uniform.

**Fig. 3.**
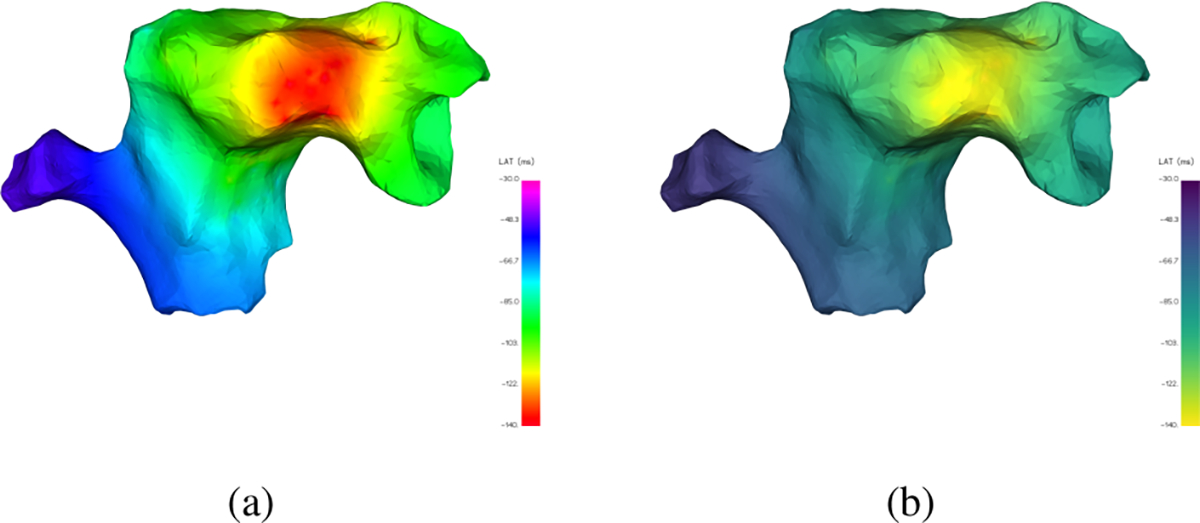
For the same LAT signal, the *gist_rainbow* colormap (a) suggests bands of rapid LAT value change (yellow and cyan regions) while the *viridis* colormap (b) reflects the smooth transitions of the underlying data.

Two problems arise when trying to quantify perceived differences. First, LAT values are assigned colors according to the chosen colormap, typically the *gist_rainbow* colormap in Fig. 2(a), which contains several “kinks” where the color changes drastically over a small range of values. These ranges are therefore far more sensitive to small changes in the LAT value and far more prone to large perceptual error. Rainbow colormaps have been heavily criticized in the literature as poor visual representations of sequential data because of this issue of non-uniformity and because of the lack of intuitive color ordering (it’s not obvious that green should be lower than purple). Generally, it is recommended to replace them with a more perceptually uniform and sequential colormap like *viridis* in Fig. 2(b) [41], [42]. As an example, consider the LAT map in Fig. 3 where the same underlying signal values which vary smoothly over the surface are represented by the *gist_rainbow* and *viridis* colormaps. The *gist_rainbow* map introduces a false perception of rapid signal transition from red to green while the *viridis* map clearly represents the true smooth signal.

**Fig. 4.**
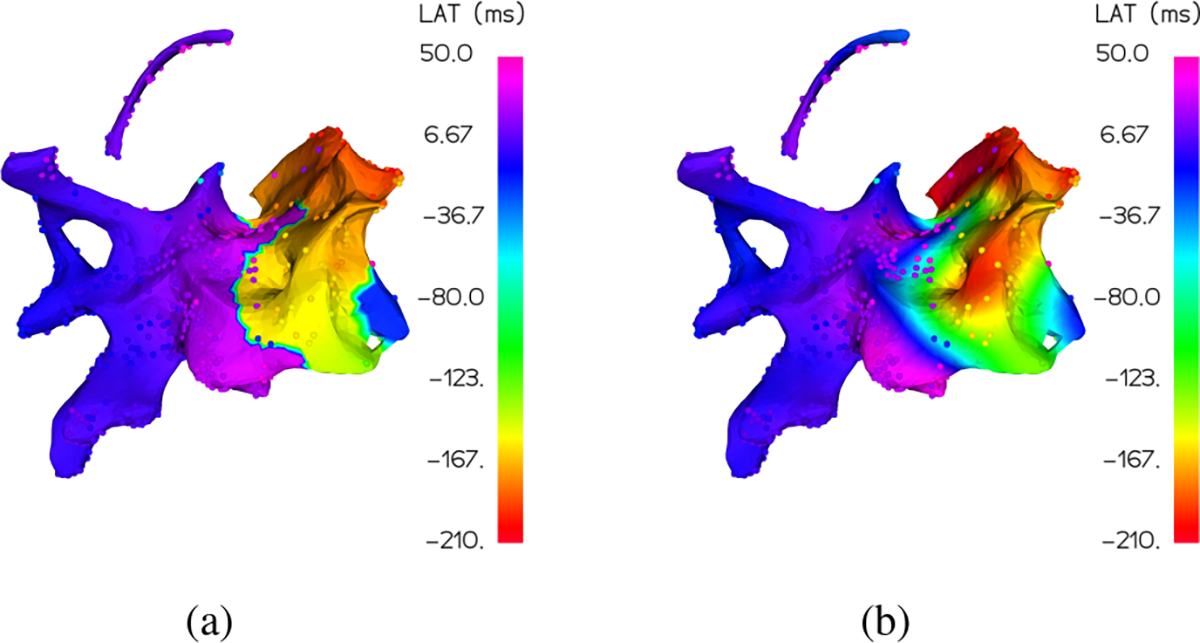
For two possible interpolations of the same LAT signal, with the interpolation coloring the surface under the ground truth points, the map in (a) is perceptually more accurate but has a higher normalized mean squared error than the map in (b).

The second issue with quantifying color differences is that human perception is in general non-uniform, so standard metrics like mean squared error (MSE) or mean absolute error (MAE) applied to either the LAT values or the red-green-blue (RGB) color channels often don’t correctly measure perceived accuracy [43]–[48]. The same LAT mapping rescaled or shifted by a small bias would result in a very large MSE while potentially being perfectly reasonable in a visual representation. On the flip side, large visual color differences can correspond to very small shifts in RGB space, resulting in a low MSE. For a practical example of this, consider the interpolated LAT map in Fig. 4(a) which visually matches the overall ground truth much better than the map in Fig. 4(b) but has a higher normalized mean square error (NMSE) of 0.21 compared to 0.18.

To solve these problems, we propose a new metric, Mean Delta-E (MDE), based on the standard ΔE* color distance metric (CIEDE2000) [37]. Given an estimated LAT value $\hat{s_{i}}$, its ground truth value *s*_*i*_, and an LAT map scale [*s*_*min*_*, s*_*max*_], we must first represent the LAT values as colors appropriate for visualization on an insightful LAT map. We therefore assign colors $\hat{c_{i}}=\left(\hat{r_{i}},\;\hat{g_{i}},\hat{\;b_{i}}\right)$ and *c*_*i*_ = (*r*_*i*_*, g*_*i*_*, b*_*i*_) to $\hat{s_{i}}$ and *s*_*i*_ respectively according to the colormap *viridis* with minimum and maximum values corresponding to [*s*_*min*_*, s*_*max*_].

Now, to measure the difference between $\hat{c_{i}}$ and *c*_*i*_, we could naively take the distance between the RGB channels, but as mentioned previously, the non-uniformity of human perception makes this a poor color distance measure. Instead, we translate the colors into the colorspace CIELAB, which mimics human perception and encodes color in terms of L* (lightness), a* (red-green opponents), and b* (blue-yellow opponents). Even this colorspace, however, is not fully perceptually uniform, so to accommodate the remaining non-linearities, we take the difference of ${\hat{c}}_{i}=\left({\hat{L}}_{i}^{*},\;{\hat{a}}_{i}^{*},\;{\hat{b}}_{i}^{*}\right)$ and $c_{i}=\left(L_{i}^{*},\;a_{i}^{*},\;b_{i}^{*}\right)$ with the ΔE* color distance equation (CIEDE2000^1^),

(3) $$\Delta E_{00}^{*}=\{{\left(\frac{\Delta L^{\prime }}{k_{L}S_{L}}\right)}^{2}+{\left(\frac{\Delta C^{\prime }}{k_{C}S_{C}}\right)}^{2}+{\left(\frac{\Delta H^{\prime }}{k_{H}S_{H}}\right)}^{2}\;{\;\;+R_{T}\left(\frac{\Delta C^{\prime }}{k_{C}S_{C}}\right)\left(\frac{\Delta H^{\prime }}{k_{H}S_{H}}\right)\}}^{1/2}$$

whereΔ*L*′, Δ*C*′, and Δ*H*′ correspond to differences in lightness, chroma, and hue respectively, with weighting factors *k*_*L*_, *k*_*C*_, *k*_*H*_, compensation factors *S*_*L*_, *S*_*C*_, *S*_*H*_, and a hue rotation term *R*_*T*_ [37]. This equation is the current standard measure for small to medium color differences and is therefore well suited to measure small errors in LAT values with respect to the *viridis* colormap. For our application, we use the default recommended hyperparameters [49].

Once we have computed the individual color differences for all interpolated points relative to the ground truth, we take the mean of the result as our overall evaluation metric MDE. Returning to the example in Fig. 4, we find that the more accurate map in 4(a) correctly has a lower MDE of 4.76 compared to 4(b) with 5.70.

MDE provides an objective similarity measure for LAT map interpolation that leverages established color theory to accurately quantify visual differences in LAT maps. These visual differences directly determine the usability of a given interpolation for ablation treatment. A more visually accurate map will result in a better ablation and can indeed be more “physically” accurate relative to the true tissue activation patterns than another less visually accurate map that exhibits a lower MSE.

Additionally, this metric is easily adaptable to focus on error in particular LAT value ranges. During ablation procedures, the colormap range is often adjusted to show greater detail for low activation times and assign all high LAT values the same color. See Fig. 1 where all values greater than −1 ms are assigned the same pink color. By simply setting the *viridis* colormap range to the value range of interest, the MDE metric will automatically assign zero error to any points with estimated and true values outside of that range and provide better error granularity for the remaining range since the new color assignments will be more differentiated. This allows researchers to easily investigate perceptual error at various scales depending on the specific LAT map application (focal point localization, arrhythmia classification, ablation target prediction, etc.).

### E. Realistic Sub-Sampling for Testing

To select a representative subset of LAT observations for interpolation testing, we should mimic the typical distribution of points that would be obtained during the mapping process. Without a larger dataset spanning many patients, multiple types of arrhythmias and ordered LAT observations, it is difficult to directly measure what would be the typical distribution of LAT observations during an incomplete mapping process. We therefore instead construct a non-uniform distribution that is easy to implement and adheres to a few simple principles defined by the mapping process itself:

1. The mapping process starts blindly and occasionally jumps to a new area, so all LAT values should have non-zero and non-trivial sampling probability i.e. no probability should be vanishingly small.
2. The mapping process generally stops when the points of earliest activation are definitely found, so these should not be selected with the highest probability.
3. The technician, while searching for the region of earliest activation, more densely samples mid- to early-activation points rather than later activation regions.

**Fig. 5.**
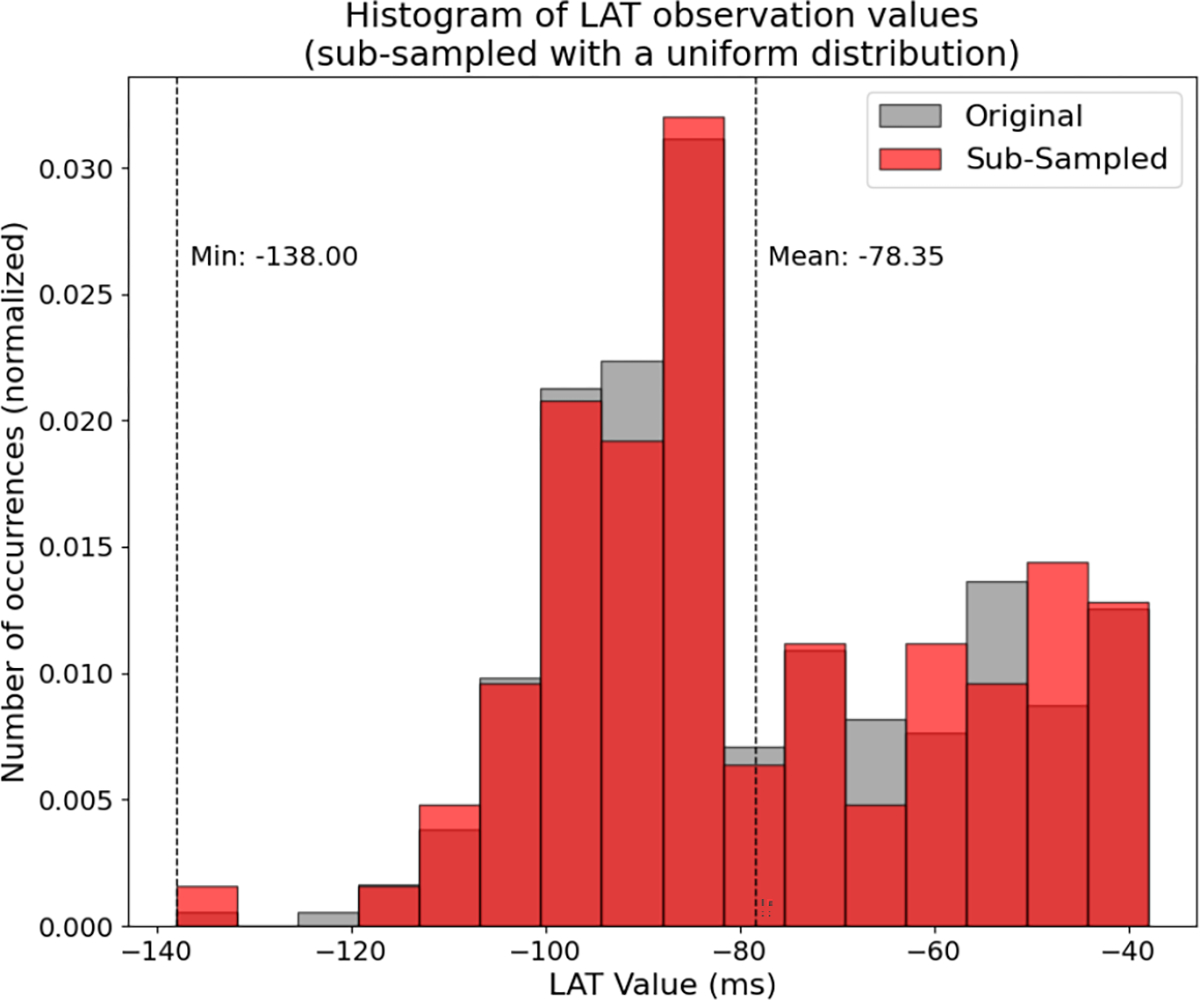
An example result (transparent red distribution) of sub-sampling LAT Map 1 (grey) with a uniform distribution. There is a much more even distribution of points across the whole range than we would expect in a clinical scenario.

To see why this is necessary, consider in Fig. 5 the distribution in red of a result of naively randomly sampling the LAT signal for Map 1 (grey) with a uniform distribution. In this case, there are relatively more low-value points selected than we would expect to see in a clinical scenario.

To avoid this, our distribution is therefore defined as follows. First, for ease of notation, we assume the LAT values are non-negative; if this does not hold, simply shift the LAT values to be all positive with *x*_*i*_ = *s*_*i*_ + |*s*_*min*_|. Then we compute the intended highest probability observation as

(4) $$x^{\prime }=x_{min}+\lambda_{1}x_{avg}.$$

Here, *λ*_1_ controls how much we favor the earliest activation points for sampling. *λ*_1_ = 0 corresponds to the earliest activation points being the most probable, which is not realistic, since usually those few are found last during the clinical mapping. *λ*_1_ = 1 corresponds to the average-value activation points being the most probable, also unlikely since technicians would more densely sample around any earlier observations that they find. We therefore choose *λ*_1_ = 0.5, estimating that the highest percentage of observations during the mapping would be between the two extremes.

Next, intuitively, we realize that LAT observations that are further from this most densely sampled value should have lower selection probability. For simplicity, we therefore choose the relative sampling probability of each observation *x*_*i*_ to be directly and negatively proportional to its difference from *x*′. We calculate the absolute distance from each *x*_*i*_ to *x*′ and construct a function *r* such that the output *r*_*i*_ is positive and negatively proportional to that distance,

(5) $$\begin{array}{c} d_{i}=\left|x_{i}-x^{\prime }\right|,\\ r\left(d_{i}\right)=\lambda_{2}\left(d_{max}-d_{i}\right)+1. \end{array}$$

Here, *d*_*max*_ = max_*i*∈[0, . . . , *m*]_ *d*_*i*_, so the minimum value of *r* is always 1, and *λ*_2_ is the proportionality constant that controls the slope of the resulting distribution. We choose the slowly decreasing slope of *λ*_2_ = 0.25 so that all observations have a non-trivial probability of selection.

To simplify implementation of this sampling protocol, we additionally cast each value to an integer,

(6) $$f_{i}=\mathrm{int}\left[r_{i}\right].$$

This allows us to directly add each observation to a list with integer repetition *f*_*i*_ and uniformly sample the result to obtain our test signal. The final sampling probability is given by

(7) $$p_{i}=\frac{f_{i}}{\sum_{i}f_{i}}.$$

**Fig. 6.**
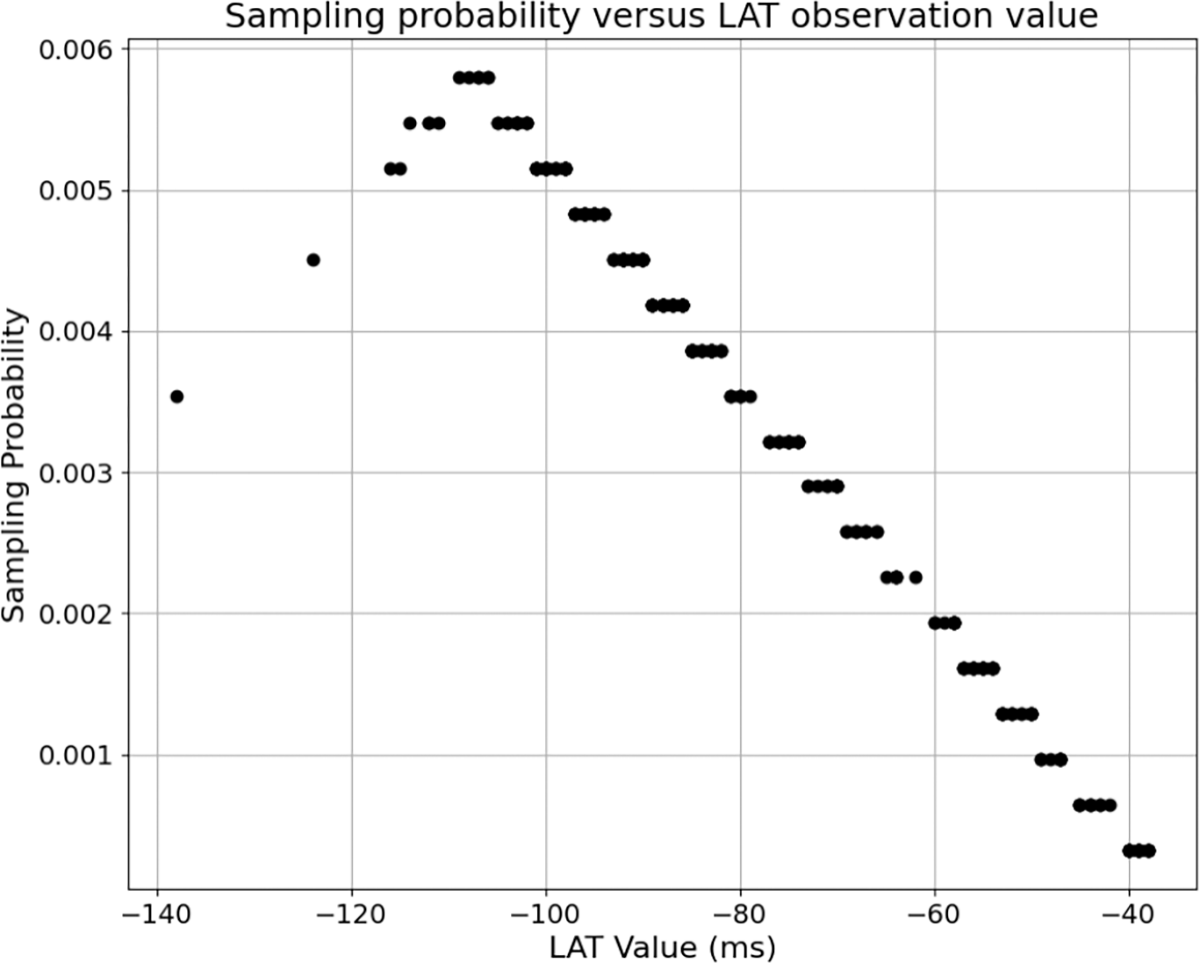
An example of the non-uniform sampling distribution used in sub-sampling for testing LAT Map 1 to mimic a probable distribution of observations in an ablation procedure.

**Fig. 7.**
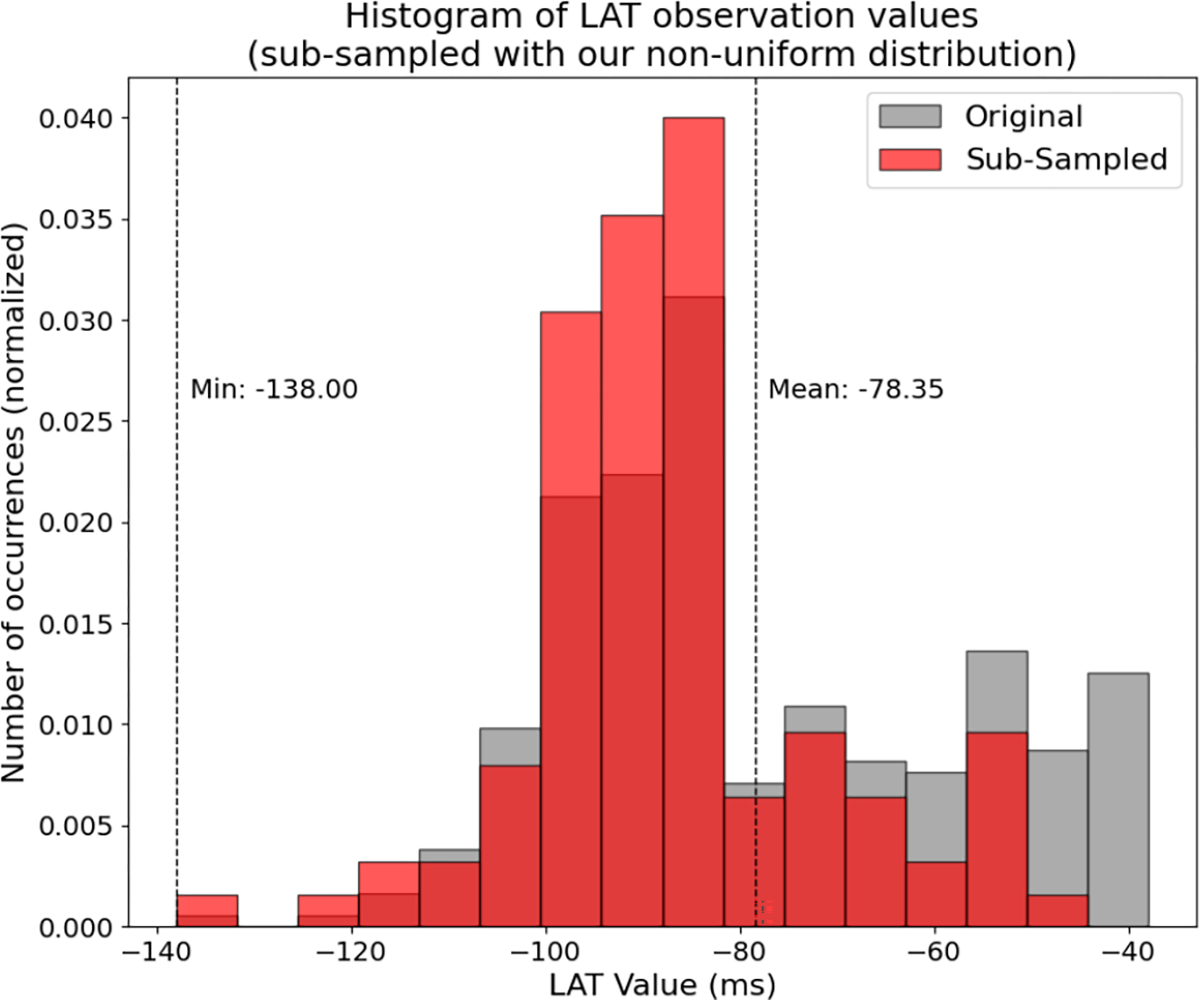
An example result (transparent red distribution) of sub-sampling LAT Map 1 (grey) with our proposed non-uniform distribution in Fig. 6. It correctly results in a distribution favoring mid- to early-activation times.

For LAT Map 1, we show this sampling distribution in Fig. 6 and an example of the distribution of the sub-sampled signal randomly selected based on that distribution in Fig. 7. The most probable observations selected for the test signal (*s*_*i*_ ≈ −110 ms) are those halfway between the earliest and average activation times. The sampling probability decreases linearly away from that value in both directions, so that the point of earliest activation has the same probability of being selected as the point of mean activation time. All later activation points have a lower but nonzero sampling probability. The resulting subset in Fig. 7 mostly has points in the early- to mid-activation range, as desired, with some later values also.

Overall, this non-uniform distribution allows us to generate random subsets of LAT observations that are similar in distribution to those found during LAT mapping in ablation procedures. More involved sub-sampling distributions based on the spatial density of samples available as well as the LAT values of those samples are certainly possible, and we discuss this more in Section VI. For this work, the proposed protocol was hand-crafted based on the suggestions of our medical colleagues and proved sufficiently realistic to be useful in evaluation.

## IV. Experimental Setup

To thoroughly test our proposed method, we implement alternative interpolation methods as described in Section IV-A, cross-validate with random selection and repetition as detailed in Section IV-B, and post-process and visualize the interpolations as outlined in Section IV-C.

### A. Implementing Prior Interpolation Algorithms

To compare performance versus other interpolation algorithms, we implement and test in parallel two alternative methods, Gaussian Process Regression (GPR) and Gaussian Process Manifold Interpolation (GPMI) [17]. GPR is a standard out-of-the-box interpolation method for data in higher dimensions; for this, we use a kernel sum of three radial basis functions with length scales of 0.01, 0.1, and 1. GPMI is state-of-the-art in the literature for LAT interpolation and defines a Gaussian process model on the manifold instead of simple Euclidean space.

### B. Cross-Validating With Random Selection and Repetition

For all maps and before processing by any interpolation algorithm, we perform the pre-processing of the LAT samples as described in Section III-A to obtain the usable set of observations ${𝒱}_{𝒮}$.

Then for random selection of *m* LAT observations, we use the non-uniform sub-sampling distribution and protocol described in Section III-E to obtain a subset ${𝒱}_{train}\subset {𝒱}_{𝒮}$ of LAT observations where $\left|{𝒱}_{train}\right|=m$. The same subset ${𝒱}_{train}$ is used as input where relevant for MAGIC-LAT, GPMI, and GPR interpolation. The complementary subset ${𝒱}_{test}\subset {𝒱}_{𝒮}$ is then compared to the interpolation results for each method, and the respective MDE errors are computed for that test iteration.

For cross-validation by repetition, we repeat the above process, generating a new random ${𝒱}_{train}$ for each test iteration. The respective MDE’s for each method are recorded, and the mean and standard deviations across all iterations are reported.

### C. Post-Processing and Visualizing Results

Each interpolation algorithm estimates the signal value at the vertices of the 3 d mesh. To color the mesh faces and visualize the final maps as in the following figures, we use the vedo Python module [50]. The included interpolation function colors the faces based on simple Shepard interpolation (inverse distance weighting) of the nearest vertex values. Note that our visual results are still be presented with the *gist_rainbow* colormap for the convenience of medical experts who are only accustomed to that representation. The same figures represented with the *viridis* colormap may be found in the attached Supplementary Document.

## V. Results

Our results are organized as follows. Section V-A validates MAGIC-LAT on the simulated left atrium case. Section V-B visually demonstrates for a few clinical patients the capability of MAGIC-LAT to interpolate only 100 observations into an accurate map. Section V-C provides cross-validated results for 100 input observations and compares MAGIC-LAT performance to that of the prior methods. Section V-D addresses cross-validated performance for varied numbers of input observations. And Section V-E examines the algorithm execution time.

### A. Cross-Validation Interpolation Results on Simulated Patient

**Fig. 8.**
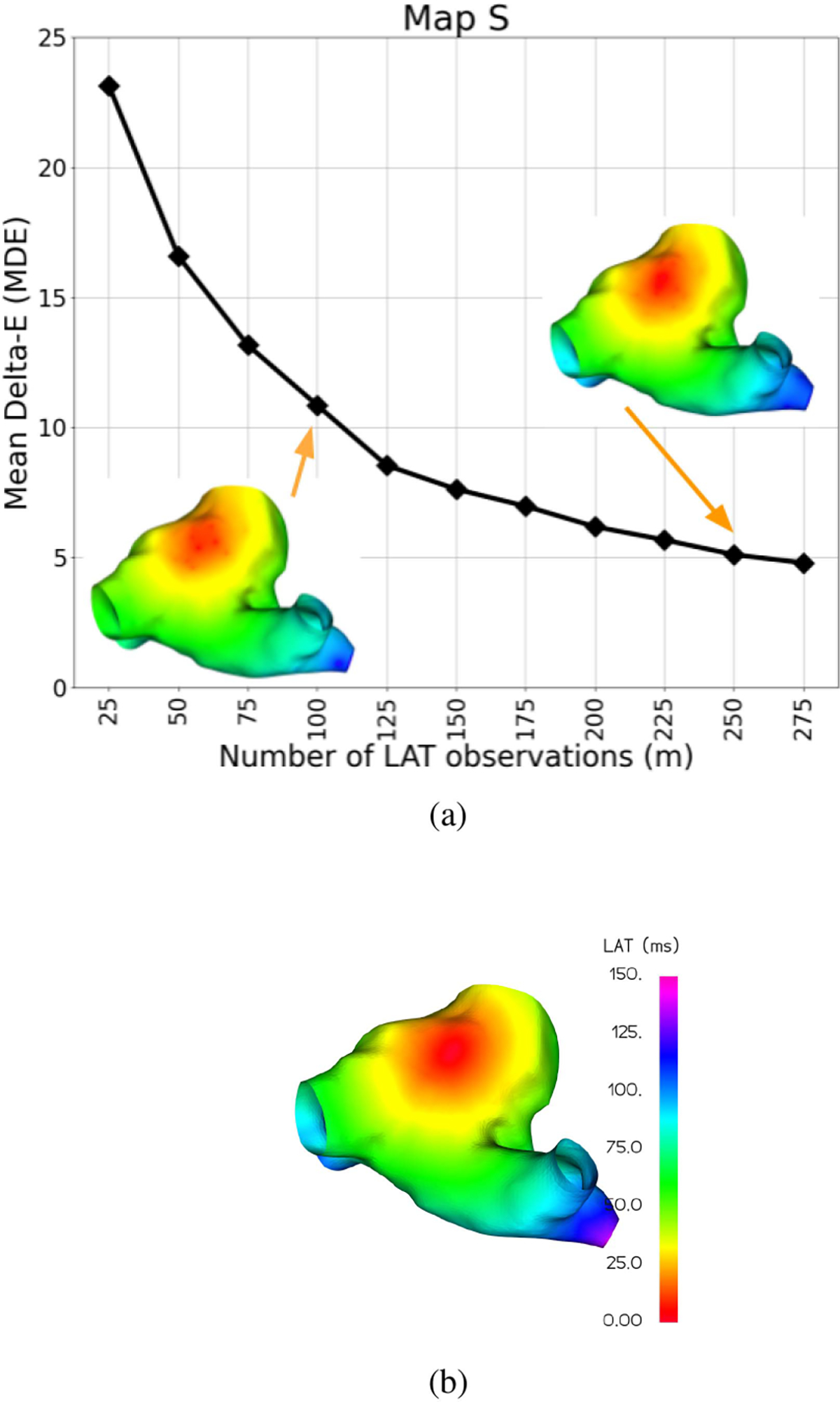
Cross-validated Mean Delta-E (MDE) results (a) for interpolation of *m* = 50–275 input LAT observations with 25 random repetitions on the simulated Map S indicate excellent map estimation for only 100 observations as compared to the complete ground truth (b).

To validate the functionality of MAGIC-LAT on a map with a complete, known ground truth, we first interpolate a varied number of input LAT observations on the simulated Map S in Fig. 8(b), with a total of 25 repetitions per test. The resulting average errors across iterations are shown in Fig. 8(a). As expected, the interpolation error initially decreases rapidly with increasing number of observations but quickly exhibits diminishing returns.

We therefore find that MAGIC-LAT is able to quite accurately estimate the key region of earliest activation (red) with only 100 input samples, and further increasing the number of input samples only improves on the finer details of the map.

### B. Visual Interpolation Results for 100 LAT Observations

**Fig. 9.**
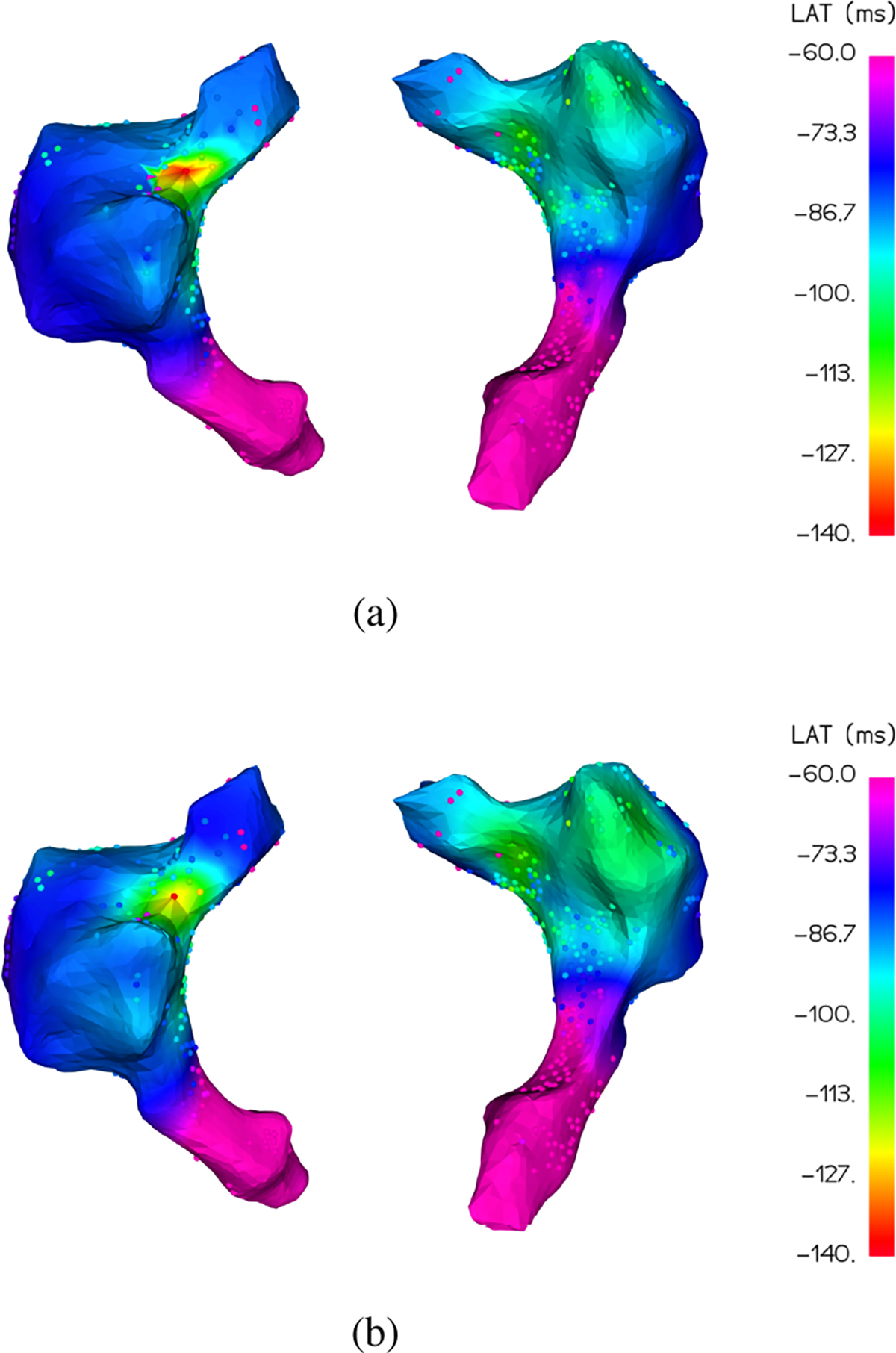
Patient A, Map 1 interpolation by MAGIC-LAT (a) and GPMI (b) for *m* = 100 input LAT observations. The interpolations color the manifold surface underneath the known LAT observations. MAGIC-LAT correctly estimates a small region of early activation (red) that GPMI does not, and has a lower MDE of 7.93 versus 8.25 for GPMI.

First, we select only *m* = 100 LAT observations as input and interpolate them across the manifold surface with the results shown in Fig. 9 for Patient A, Map 1. In Fig. 9(a), the MAGIC-LAT interpolation result closely approximates the ground truth values overlayed as points on the surface, achieving an MDE of 7.93 using only 34% of the available points. It correctly estimates a small region of early activation (red) that GPMI in Fig. 9(b) does not, making it a better map for choosing ablation targets. Indeed, this early activation region aligns well with the ultimate ablation targets as shown in Fig. 1 of the Supplementary Document.

**Fig. 10.**
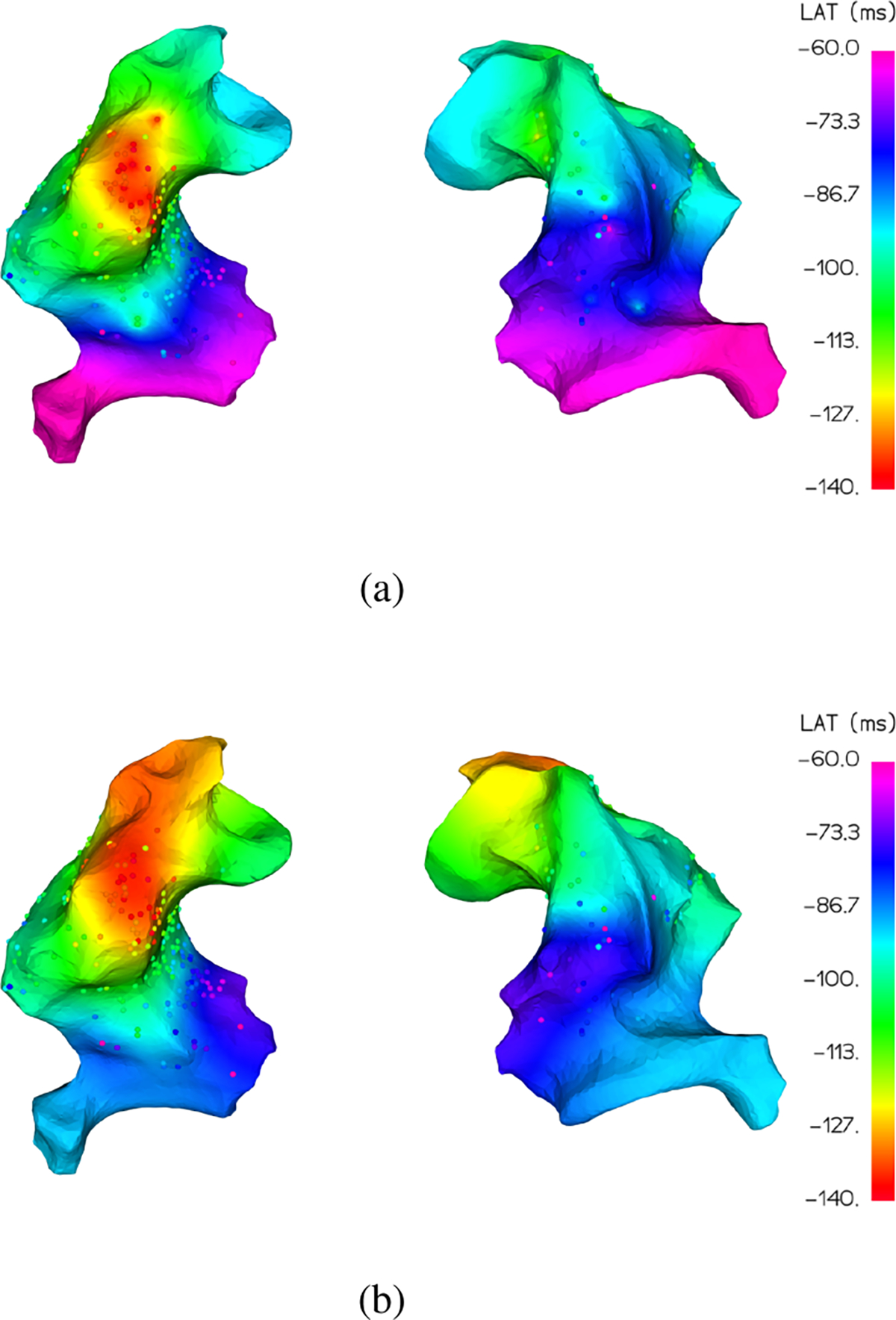
Patient B, Map 3 interpolation by MAGIC-LAT (a) and GPMI (b) for *m* = 100 input LAT observations. The interpolations color the manifold surface underneath the known LAT observations. MAGIC-LAT estimates a smaller region of early activation (red) and extrapolates the signal out to lower values (purple) than GPMI, resulting in a lower MDE of 7.99 versus 8.75 for GPMI.

For the same experiment on Map 3 for Patient B, we see similar results in Fig. 10 where the MAGIC-LAT interpolation achieves a lower MDE compared to GPMI (7.99 versus 8.75) for 100 points, only 42% of all available samples. In this case, however, GPMI estimates a very large region of early activation that would probably not be useful for choosing ablation targets. MAGIC-LAT instead estimates a relatively small region that matches up well with the final ablation targets that successfully halted the patient’s arrhythmia, shown in Fig. 2 of the Supplementary Document. In addition, MAGIC-LAT correctly extrapolates the signal out to lower values (purple) than GPMI on the bottom part of the map.

Note that both of these maps (1 and 3) correspond to patients experiencing a PVC, which is a type of focal arrhythmia. Focal arrhythmias originate in a single location with a centrifugally expanding wavefront. In these cases, identification of a discrete focus and not a broader swath of early activation is of paramount importance. This allows for pinpoint targeting of the culprit region and narrows the ablation target, minimizing the amount of unnecessary ablation energy delivery and by extension tissue damage. For both patients, MAGIC-LAT captures the tight focal area of early activation where GPMI fails to do so.

**Fig. 11.**
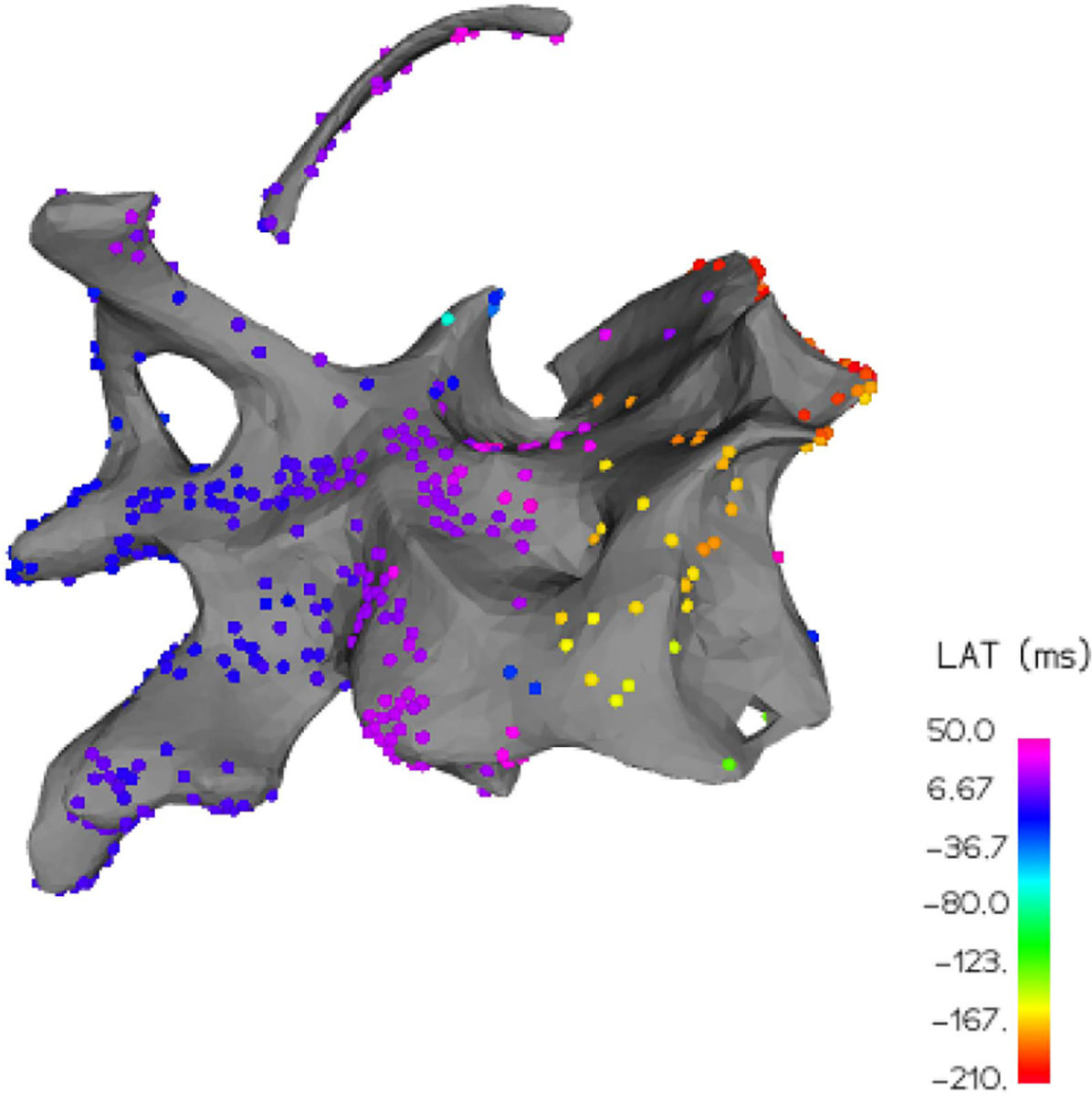
LAT Map 6 exhibits an “early-meets-late” region of high frequency variation which is challenging to accurately interpolate.

Among the patients, the LAT map for patient D holds an interesting and distinctive feature, a sizable spatial region of drastic signal variation which is visible in Fig. 11, where vertices with very low LAT values (e.g. −200ms) are adjacent to vertices with high LAT values (e.g. +50ms). This “early-meets-late” phenomenon can occur in patients with macro re-entrant circuits overriding the natural sinus rhythm and causing arrhythmia. In these types of circuitous (i.e. non-focal) arrhythmias where there is no discrete early activation region, regions identified as “early-meets-late” may represent isthmuses of delayed conduction that provide the substrate for arrhythmia. These regions are therefore targeted for ablation. The “early-meets-late” phenomenon can also occur when the reference for measuring local activation time is changed or disturbed, so that some LAT samples are measured relative to a different reference. It can even occur, as it did in this case, when the chosen cycle window is too narrow, so that very late activation regions are incorrectly tagged as early activation under the assumption that a second heartbeat has occurred. Regardless, this local high-frequency content breaks the typical smoothness assumption of our signal, making the interpolation quite difficult.

**Fig. 12.**
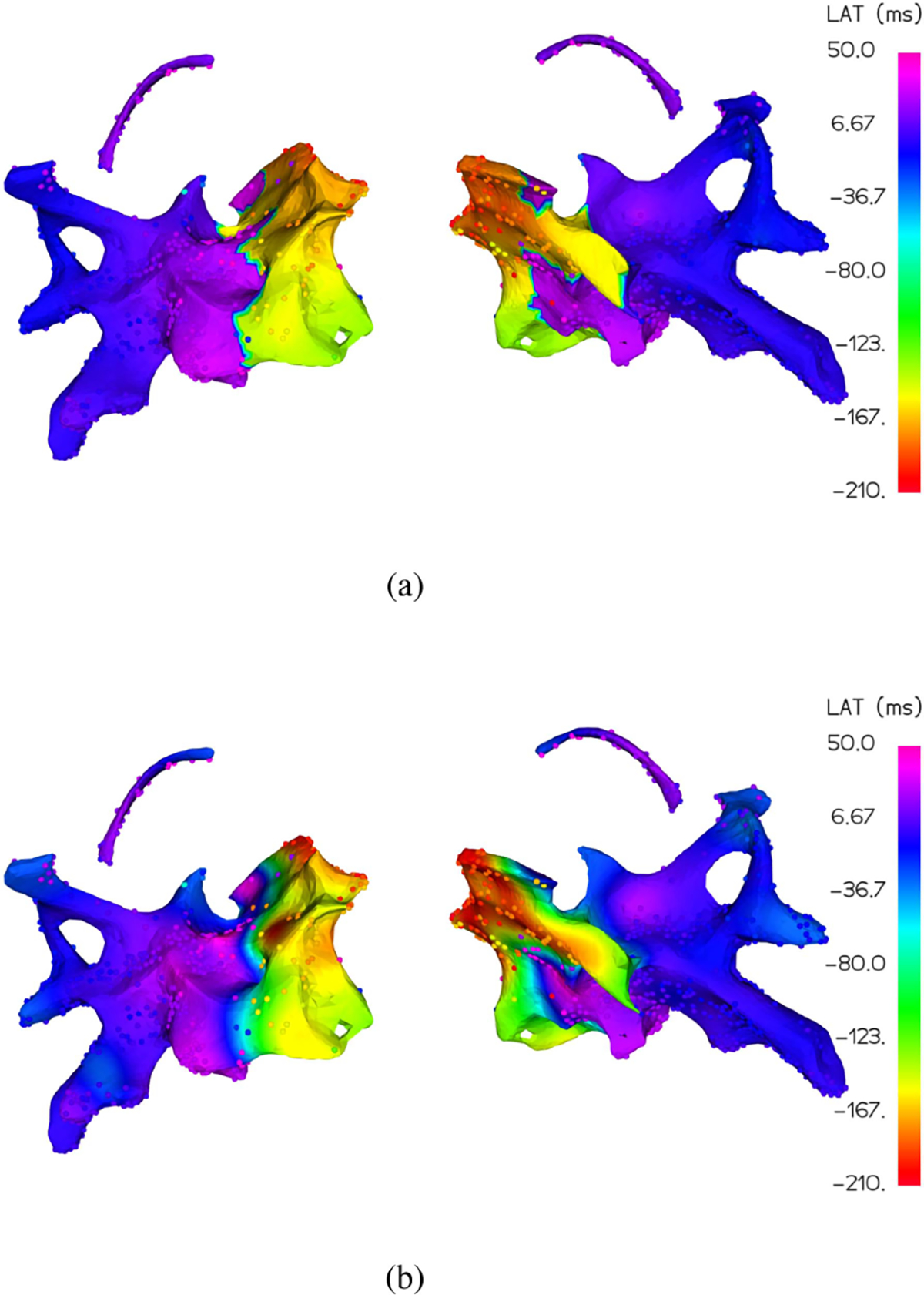
Patient D, Map 6 interpolation by MAGIC-LAT (a) and GPMI (b) for *m* = 100 input LAT observations. The interpolations color the manifold surface underneath the known LAT observations. MAGIC-LAT very closely approximates the sharp early-meets-late transition region, while GPMI erroneously smooths it out with a wide blue to yellow transition band.

For this map, we show the interpolation results for MAGIC-LAT and GPMI in Fig. 12 using 100 sample points, only 14% of all available. GPMI assumes a sort of smooth Gaussian prior on the data, and as a result, filters out the underlying sharp transition in the data. It loses the distinct “early-meets-late” region, a critical piece of information, and therefore could lead to an improper treatment, possibly even a misplaced ablation. Our method, however, accommodates the local signal jump by updating the graph structure based on given observations and generates a better LAT map with a correspondingly lower MDE of 4.67 compared to 6.62 for GPMI.

In general, a more distinct interpolation feature (either a discrete focal area of early activation or a clear “early-meets-late” boundary) provides a more precise ablation target, minimizing excess ablation and any risk of collateral damage to the patient. From an application perspective, these examples demonstrate the capability of MAGIC-LAT to estimate from relatively few observations the key LAT features for arrhythmia diagnosis and ablation treatment. From a signal processing perspective, they demonstrate the flexibility of MAGIC-LAT to accurately interpolate both smooth and locally discontinuous LAT observations.

### C. Cross-Validation Interpolation Results for 100 LAT Observations

We repeat the same experiment 50x on all patient maps for all methods and record the average MDE and standard deviations in Table III, including the corresponding NMSE of LAT values for further validation. Our method consistently outperforms GPR and GPMI in all cases, and in particular performs significantly better on Map 6, where it accurately interpolates the difficult early-meets-late signal feature. In the case of Map 4, for which MDE across all methods is noticeably higher, we found that the LAT observations were unusually noisy with patchy discontinuities even though the patient was in normal sinus rhythm, making the interpolation task more difficult.

### D. Cross-Validation Interpolation Results for Varied Sizes of Input LAT Observations

For our final experiment, we vary the number of input LAT observations and repeat the previous cross-validation, reducing the number of runs to 25 for each value. Results are summarized graphically for all methods on all maps in Fig. 3 of the Supplementary Document. Note that values for *m* = 100 observations may differ slightly from those listed in Table III due to the higher variance of fewer iterations. GPR, the out-of-the-box benchmark, shows poor performance overall. MAGIC-LAT consistently outperforms GPR and GPMI in almost all cases with the exception of very low numbers of input observations (50–100) for Maps 2 and 3, where GPMI achieves lower MDE. MAGIC-LAT is also significantly better for Map 6, as expected.

### E. Complexity and Optimization Considerations

The complexity and speed of MAGIC-LAT is primarily limited by the matrix inversion operation which is *O*(*n*^3^), where n=|𝒱|, the number of graph vertices. The timing results for executing MAGIC-LAT over 50 iterations on an Intel i5 CPU with 8 GB RAM with many background processes are summarized in Table 1 of the Supplementary Document along with the corresponding graph size. Even with no specific optimization and average workstation specs, the method executes in at most 10 seconds for the larger maps and less than 2 seconds for the smaller. Therefore, on a dedicated machine with more RAM, this implementation could likely be used as-is for all but the largest meshes.

Moreover, many software packages exist to accelerate linear algebra operations [51], and numerous groups have implemented matrix inversion hardware accelerators in various contexts [52], [53]. Since MAGIC-LAT is based on such fundamental and popular operations, a dedicated machine running a hardware- and software-optimized version of the method would very reasonably achieve near real-time performance for use in ablation procedures.

## VI. Discussion

These experiments are limited by the number of available ground truth measured LAT observations, as all but two maps contain less than 350 measurements. Nevertheless, we see that our method generates accurate interpolated LAT maps using only 100 observations. Implementation of this method would undoubtedly speed up the majority of cardiac ablation procedures, thereby reducing medical costs and resulting in better patient outcomes.

Beyond its functionality, however, MAGIC-LAT also provides nice intuition as to its underlying operation. To interpret our method, we consider the case where *α* = *β* = 1 and substitute the spectral decomposition of the Laplacian **L** = **UΛU**^*T*^ to re-write (2) as

(8) $$f^{*}=U{\left(I+\Lambda \right)}^{-1}U^{T}f_{s}.$$

To analyze (8), we recall that in Euclidean space, the Laplace operator denoted as ∇^2^, is defined as the divergence of the gradient and is given by the sum of all unmixed second partial derivatives, $\nabla^{2}f=\sum_{i}\frac{\partial^{2}f}{\partial x_{i}^{2}}$, for Cartesian coordinates *x*_*i*_. The Laplacian appears in differential equations describing heat diffusion, wave propagation, and other physical phenomena governed by second-order mechanics. We see that eigenfunctions of the Laplacian form a Fourier basis since we have

$$\nabla^{2}e^{2\pi iwt}=-{\left(2\pi w\right)}^{2}e^{2\pi iwt}.$$

The generalization of the Laplacian to functions defined on Riemannian manifolds is the Laplace-Beltrami operator. Interestingly, the graph Laplacian converges to the Laplace-Beltrami operator under certain conditions (see [33], [34]), and Laplacian eigenvectors therefore form a good approximation of a Fourier basis on the manifold. This gives us the notion of a Fourier transform of a graph signal given by $\hat{f}=U^{T}f$ with inverse transform $f=U\hat{f}$. By extension, a general filtration of **f** with filter **h** is

(9) $$h(f)=U\hat{h}(\Lambda )U^{T}f,$$

where $\hat{h}(\Lambda )=diag\left(\hat{h}\left(\lambda_{1}\right),\ldots ,\hat{h}\left(\lambda_{n}\right)\right)$ is the spectral representation of the filter [29].

Comparing (8) with (9), we immediately observe that our interpolation solution is the output of a filter on our input signal **f**_**s**_ with the particular filter $\hat{h}(\Lambda )={\left(I+\Lambda \right)}^{-1}$ determined by our optimization formulation. This interpolation via filtering is a novel perspective and motivates further investigation into other filter constructions. In the field of graph signal processing, there are a number of interesting works around the idea of localized graph filters [54], diffusion wavelets [55], wavelet-based semi-supervised learning [56], and wavelet neural networks [57], [58] as well as the possibility of learning the underlying graph structure [59] for problems similar to that of LAT interpolation. Extensions of our framework to incorporate these concepts could give better performance and new insight into patterns of electrical activation propagation in tissue.

One additional possible improvement on our method is suggested by our new metric MDE. The optimization formulation in (1) that gives us our solution defines signal assignment loss (the first term) as the MSE of the LAT values. As discussed previously, MSE can sometimes give poor estimations of LAT accuracy. A similar formulation that incorporates MDE instead and still allows for a closed-form solution would therefore likely result in a better interpolation.

In a similar vein, direct interpolation of the LAT colors [60] on the manifold rather than the LAT values also presents interesting possibilities. Estimating a 3-dimensional color signal on the graph would increase the complexity of the method but could potentially improve performance.

The manual removal of locally anomalous samples described in Section III-A should in the future be replaced by a robust automated process, as this is a critical pre-processing step for accurate LAT interpolation. Excluding these points essentially removes noisy data from the training subset, improving the final interpolation, and lowers the measured error on the test subset since un-physical samples are extremely difficult to estimate.

Additionally, as mentioned previously, identification of the region of earliest activation is typically most important for correct ablation treatment. As defined, our MDE metric evenly weights the error of all test points regardless of LAT value. One interesting alternative to this would be to define a weighted MDE that penalizes error in early activation test points more than late. Visually, we’ve found that our MAGIC-LAT interpolation is quite accurate in estimating this region of interest, so it is likely that the current MDE actually underestimates its performance and a weighted MDE metric would properly capture that aspect.

Finally, the sampling protocol defined in Section III-E can also be improved upon. Clinical LAT maps vary widely in the distribution of LAT values and in the spatial distribution of LAT samples on a given map. Currently, our sub-sampling method defines a distribution based only on the former because they are raw values easily manipulated into a reasonable distribution. The latter spatial density information, although more difficult to define and derive, also characterizes a realistic map, so development of a protocol that incorporates the local spatial density of samples would further improve sub-sampled LAT maps.

## VII. Conclusion

We have proposed and validated a novel method for spatial interpolation of the local activation time (LAT) signal derived during cardiac ablation procedures. We leverage the capabilities of graph signal processing to take into account the manifold surface structure underlying the LAT signal and update our representation of that structure by learning from the given LAT observations. A reformulation of the interpolation problem into a semi-supervised learning framework gives us a well-defined and insightful solution that motivates further investigation into the use of graph filtering techniques to better characterize electrical activation propagation and spatial localization in cardiac tissue. We also defined a useful sub-sampling protocol for LAT interpolation testing. For quantifying LAT map similarity i.e. interpolation error, we motivated and proposed a new metric, MDE, which incorporates established color representation and color difference theory to accurately measure the perceived differences between two LAT maps. We demonstrated that our method, MAGIC-LAT, accurately reconstructs a smooth LAT map from only 100 LAT observations and consistently outperforms existing interpolation techniques. This approach therefore shows excellent potential to reduce the average patient time in ablation surgery by decreasing the number of LAT observations needed to construct an accurate LAT map and by improving the choice of ablation targets with a more useful map.

## Supplementary Material

supp1-3166447

## Figures and Tables

**TABLE I:** Dataset Summary

Map Index	Patient	PVC (Y/N)	*n* (mesh vertices)	*m* (LAT observations)

S	-	N	11352	11352

0	A	Y	2616	307
1		Y	2616	293

2	B	Y	6103	197
3		Y	6103	240
4		N	6103	406

5	C	N	4232	326

6	D	N	6376	737

**TABLE II:** Summary of Key Notations

Notation	Definition
a, **a**, **A**	Scalar, vector, matrix
${\mathbb{R}}^{n}$	n-dimensional Euclidean spac
𝒱,ℰ,ℱ	Vertices and edges of a graph, faces of a mesh VCR $𝒱\subset {\mathbb{R}}^{3}$, ℰ⊂𝒱×𝒱, ℱ⊂𝒱×𝒱×𝒱
*w_ij_*, **W**	Adjacency matrix of a graph
**L**	Cotan-based graph Laplacian
**f**	LAT function/signal on vertices of a graph
$\hat{f}$	Fourier transform of **f**
${𝒱}_{𝒮}$	Set of sampled graph vertices ${𝒱}_{𝒮}\subset 𝒱$
**f**_**s**_	Partially sampled LAT signal
*n*	Number of vertices, $\left|𝒱\right|$
*m*	Number of LAT samples, $\left|{𝒱}_{𝒮}\right|$

**TABLE III:** Mean and Standard Deviation MDE and NMSE Results for Interpolation With *m* = 100 Input LAT Observations and 50 Random Repetitions. MAGIC-LAT Consistently Outperforms Prior Methods Across the Whole Patient Dataset

	**MDE**						

	Map 0	Map 1	Map 2	Map 3	Map 4	Map 5	Map 6

GPR	12.9 ± 1.18	11.4 ± 0.994	11.1 ± 1.04	12.2 ± 0.901	12.8 ± 0.649	6.76 ± 0.361	12.8 ± 1.11
GPMI [17]	9.66 ± 1.02	8.56 ± 0.976	8.62 ± 0.777	8.92 ± 0.470	11.7 ± 0.656	5.59 ± 0.537	6.36 ± 0.708
**MAGIC-LAT (our)**	**9.34 ± 0.668**	**8.04 ± 0.559**	**8.06 ± 0.616**	**8.86 ± 0.492**	**11.2 ± 0.575**	**5.42 ± 0.319**	**5.21 ± 0.860**

	**NMSE**						

	Map 0	Map 1	Map 2	Map 3	Map 4	Map 5	Map 6

GPR	0.40 ± 0.07	0.39 ± 0.06	0.41 ± 0.07	0.24 ± 0.03	0.48 ± 0.04	0.43 ± 0.04	0.40 ± 0.05
GPMI [17]	0.25 ± 0.08	0.24 ± 0.07	0.34 ± 0.10	0.16 ± 0.02	0.43 ± 0.04	0.39 ± 0.15	**0.22 ± 0.06**
**MAGIC-LAT (our)**	**0.22 ± 0.05**	**0.21 ± 0.04**	**0.23 ± 0.03**	**0.15 ± 0.02**	**0.42 ± 0.03**	**0.34 ± 0.034**	0.23 ± 0.07

## Footnotes

1 The Δ*E** symbol with no subscript often refers to the 1976 definition of the Δ*E** distance measure. We instead use the most recent definition published in 2000, denoted by the name CIEDE2000 and the subscript “00.”
