# Supplementary material for: Manifold Approximating Graph Interpolation of Cardiac Local Activation Time
Source: IEEE Trans Biomed Eng. Author manuscript; Available in PMC 2022 Oct 10. (PMC9549513; doi:10.1109/TBME.2022.3166447)
Supplement: supp1-3166447 [file NIHMS1837381-supplement-supp1-3166447.pdf]

Manifold Approximating Graph Interpolation of Cardiac Local Activation Time: Supplementary Document

1 Open-Source Code

All code and test scripts for this work can be found at https://github.com/jenniferhellar/magic-lat.

2 Proof of (2)

Let $\mathbf{M_l}$ and $\mathbf{M_u}$ be $n \times n$ diagonal binary matrices and $\mathbf{f}$, $\mathbf{f}^*$, and $\mathbf{f_s}$ be $n \times 1$ vectors. We define $\mathbf{f_s}$ and $\mathbf{M_l}$ such that $\mathbf{M_l f_s} = \mathbf{f_s}$. Then the solution to

$$\mathbf{f}^* = \arg\min_{\mathbf{f} \in \mathbb{R}^n} ||\mathbf{M_l}(\mathbf{f} - \mathbf{f_s})||_2^2 + \alpha||\mathbf{M_u f}||_2^2 + \beta \mathbf{f}^T \mathbf{L f},$$

is

$$\mathbf{f}^* = (\mathbf{M_l} + \alpha \mathbf{M_u} + \beta \mathbf{L})^{-1} \mathbf{f_s}.$$

Proof. To prove this, we take the partial derivative of the optimization argument with respect to $\mathbf{f}$, set it equal to 0, and solve for $\mathbf{f}$.

$\mathbf{M_l}$ and $\mathbf{M_u}$ are diagonal binary matrices therefore

$$\begin{aligned} \mathbf{M_l}^T &= \mathbf{M_l}, \qquad & \mathbf{M_l}^T \mathbf{M_l} &= \mathbf{M_l}, \\ \mathbf{M_u}^T &= \mathbf{M_u}, \qquad & \mathbf{M_u}^T \mathbf{M_u} &= \mathbf{M_u}. \end{aligned}$$

We first take the derivative of the first optimization term,

$$\begin{aligned} \frac{\partial}{\partial \mathbf{f}}\{||\mathbf{M_l}(\mathbf{f} - \mathbf{f_s})||_2^2\} &= \frac{\partial}{\partial \mathbf{f}}\{(\mathbf{M_l f} - \mathbf{M_l f_s})^T(\mathbf{M_l f} - \mathbf{M_l f_s})\} \\ &= \frac{\partial}{\partial \mathbf{f}}\{\mathbf{f}^T \mathbf{M_l}^T \mathbf{M_l f} - \mathbf{f}^T \mathbf{M_l}^T \mathbf{M_l f_s} \\ &\qquad - \mathbf{f_s}^T \mathbf{M_l}^T \mathbf{M_l f} - \mathbf{f_s}^T \mathbf{M_l}^T \mathbf{M_l f_s}\} \\ &= \frac{\partial}{\partial \mathbf{f}}\{\mathbf{f}^T \mathbf{M_l f} - \mathbf{f}^T \mathbf{M_l f_s} - \mathbf{f_s}^T \mathbf{M_l f}\} \\ &= \frac{\partial}{\partial \mathbf{f}}\{\mathbf{f}^T \mathbf{M_l f} - 2\mathbf{f}^T \mathbf{M_l f_s}\} \\ &= 2\mathbf{M_l}(\mathbf{f} - \mathbf{f_s}) \\ &= 2(\mathbf{M_l f} - \mathbf{f_s}). \end{aligned}$$

Likewise, the derivative of the second term,

$$\begin{aligned} \frac{\partial}{\partial \mathbf{f}}\{\alpha||\mathbf{M_u f}||_2^2\} &= \alpha \frac{\partial}{\partial \mathbf{f}}\{\mathbf{f}^T \mathbf{M_u}^T \mathbf{M_u f}\} \\ &= \alpha \frac{\partial}{\partial \mathbf{f}}\{\mathbf{f}^T \mathbf{M_u f}\} \\ &= 2\alpha \mathbf{M_u f}. \end{aligned}$$

And finally, the derivative of the third term,

$$\frac{\partial}{\partial \mathbf{f}}\{\beta \mathbf{f}^T \mathbf{L f}\} = 2\beta \mathbf{L f}.$$

We then combine these results, set the sum equal to 0, and solve for $\mathbf{f}$:

$$\begin{aligned} 2(\mathbf{M_l}\mathbf{f}^* - \mathbf{f_s}) + 2\alpha\mathbf{M_u}\mathbf{f}^* + 2\beta\mathbf{L}\mathbf{f}^* &= 0 \\ (\mathbf{M_l} + \alpha\mathbf{M_u} + \beta\mathbf{L})\mathbf{f}^* &= \mathbf{f_s} \\ \mathbf{f}^* &= (\mathbf{M_l} + \alpha\mathbf{M_u} + \beta\mathbf{L})^{-1}\mathbf{f_s}. \end{aligned}$$

□

3 Detailed Timing Results

Table 1: Mean and standard deviation timing results for MAGIC-LAT over 50 iterations with corresponding graph sizes.

	$n = \lvert\mathcal{V}\rvert$	runtime (s)
Map 0	2616	1.31 ± 0.15
Map 1	2616	1.26 ± 0.07
Map 2	6103	7.55 ± 0.38
Map 3	6103	7.78 ± 0.53
Map 4	6103	8.07 ± 0.72
Map 5	4232	3.36 ± 0.28
Map 6	6376	8.67 ± 0.81

4 Supplementary Figures with Ablation Targets

The following figures show the final ablation targets that halted patient arrythmias on top of the MAGIC-LAT interpolation result for the experiments described in Section V.B. of the main document.

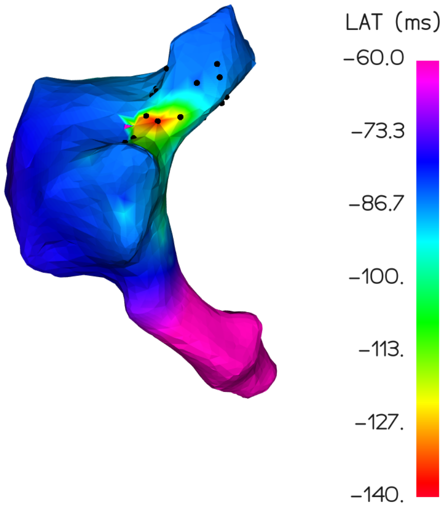

Figure 1: Patient A, Map 1 ablation targets (black) align well with the early activation region (red) estimated by MAGIC-LAT.

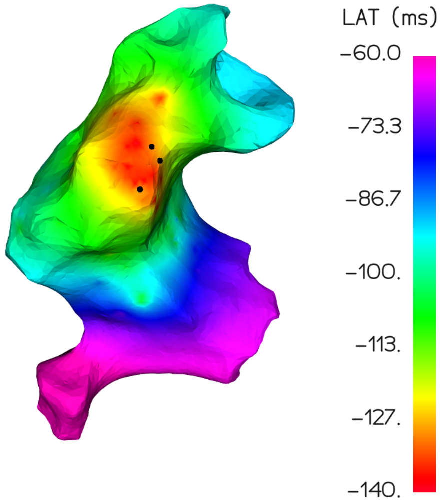

Figure 2: Patient B, Map 3 final ablation targets (black) align well with the early activation region (red) estimated by MAGIC-LAT.

5 Cross-validation results for varied sizes of input observations

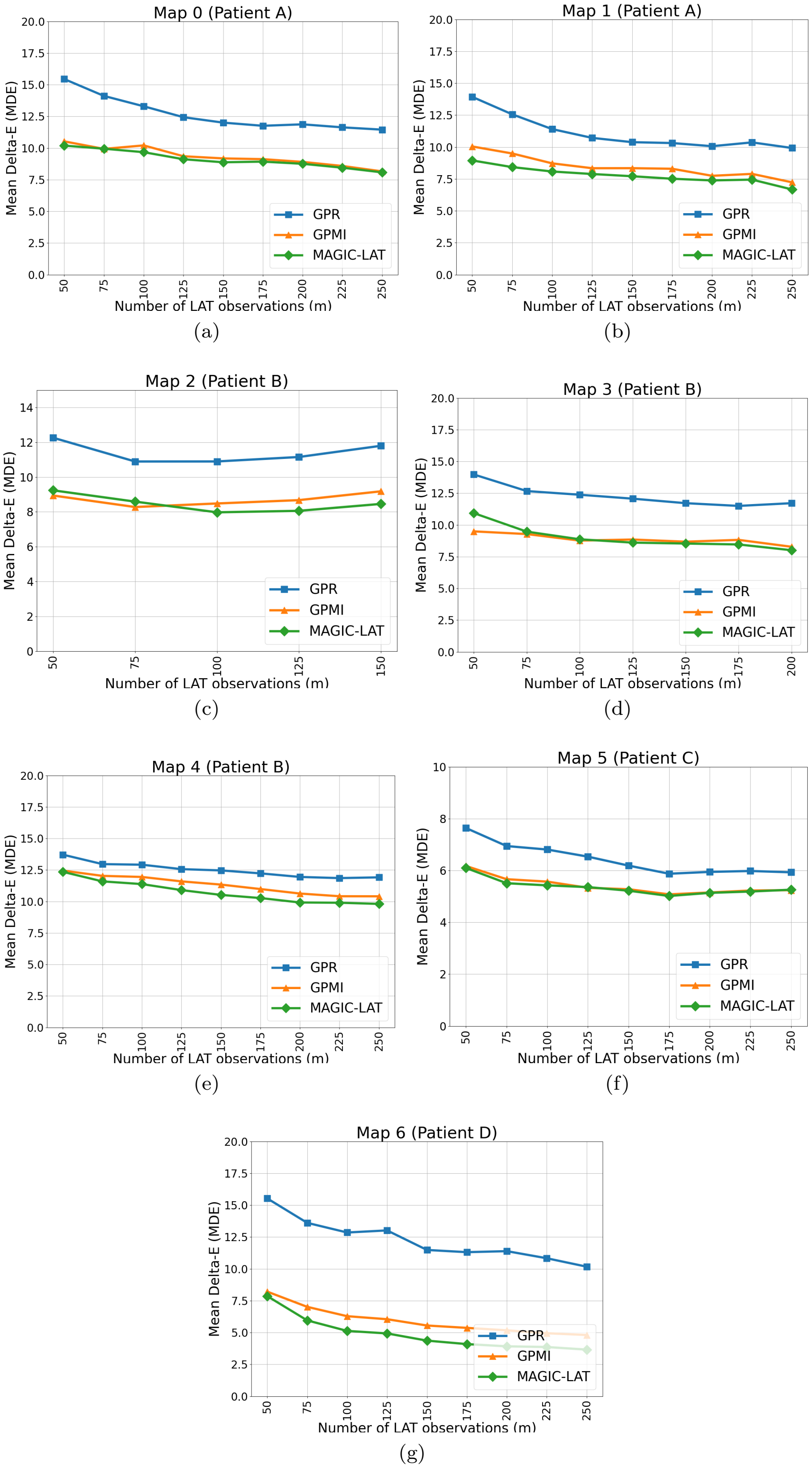

Figure 3: Cross-validated Mean Delta-E (MDE) results for interpolation of m = 50-250 input LAT observations with 25 random repetitions indicate consistently superior performance for MAGIC-LAT.

6 *viridis* colormap representation of all visual results

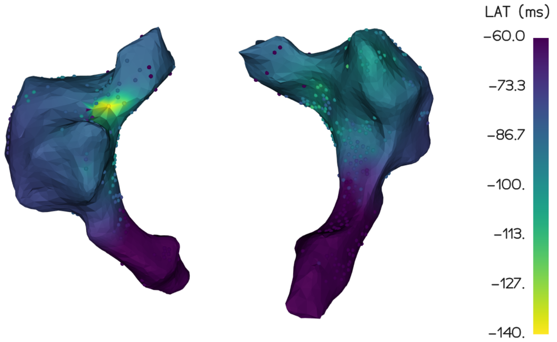

(a)

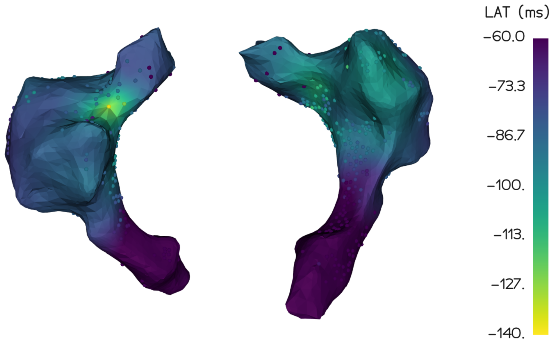

(b)

Figure 4: Patient A, Map 1 interpolation by MAGIC-LAT (a) and GPMI (b) for $m = 100$ input LAT observations. The interpolations color the manifold surface underneath the known LAT observations. MAGIC-LAT correctly estimates a small region of early activation (yellow) that GPMI does not, and has a lower MDE of 7.93 versus 8.25 for GPMI.

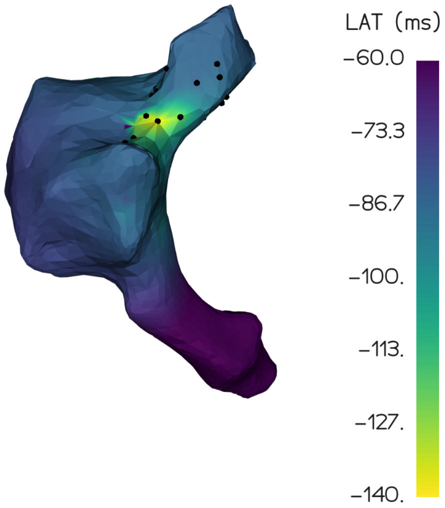

Figure 5: Patient A, Map 1 ablation targets (black) align well with the early activation region (yellow) estimated by MAGIC-LAT.

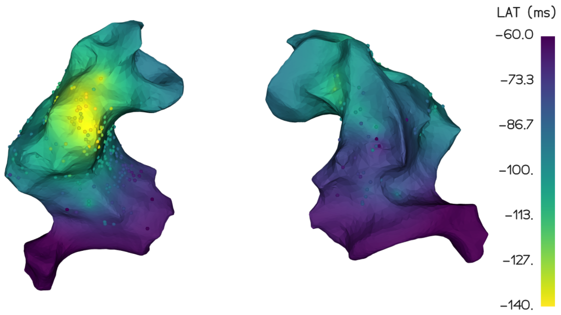

(a)

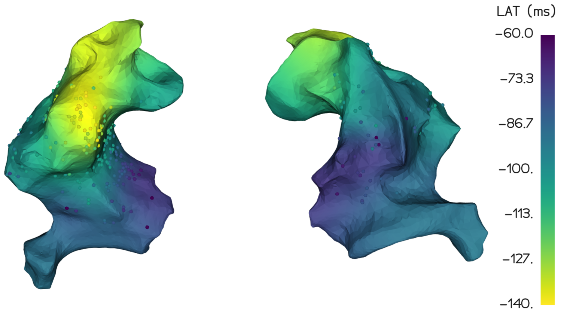

(b)

Figure 6: Patient B, Map 3 interpolation by MAGIC-LAT (a) and GPMI (b) for $m = 100$ input LAT observations. The interpolations color the manifold surface underneath the known LAT observations. MAGIC-LAT estimates a smaller region of early activation (yellow) and extrapolates the signal out to lower values (purple) than GPMI, resulting in a lower MDE of 7.99 versus 8.75 for GPMI.

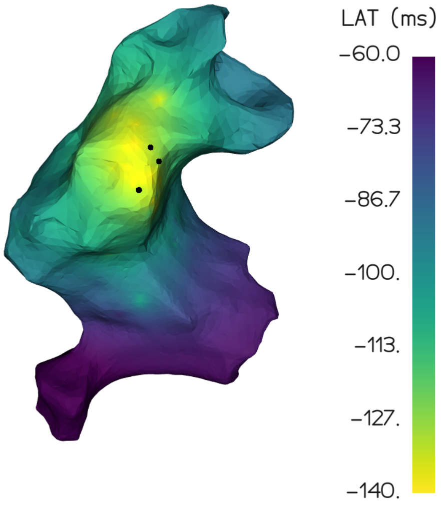

Figure 7: Patient B, Map 3 final ablation targets (black) align well with the early activation region (yellow) estimated by MAGIC-LAT.

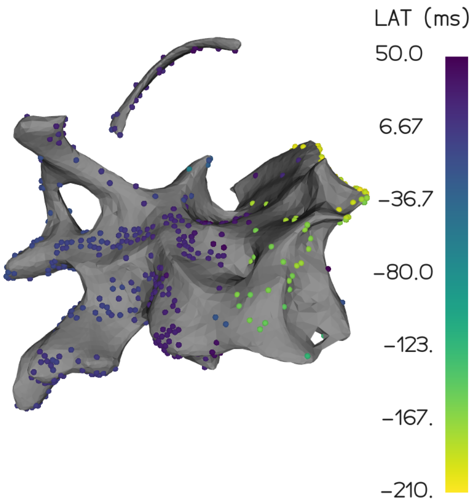

Figure 8: LAT Map 6 exhibits an “early-meets-late” region of high frequency variation which is challenging to accurately interpolate.

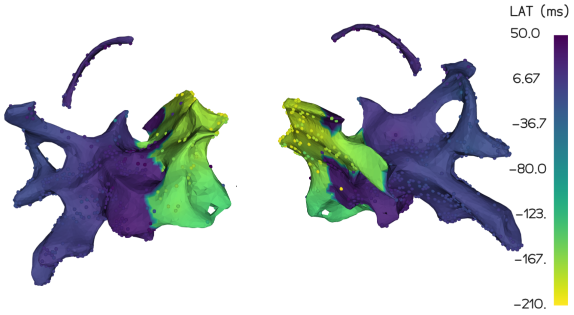

(a)

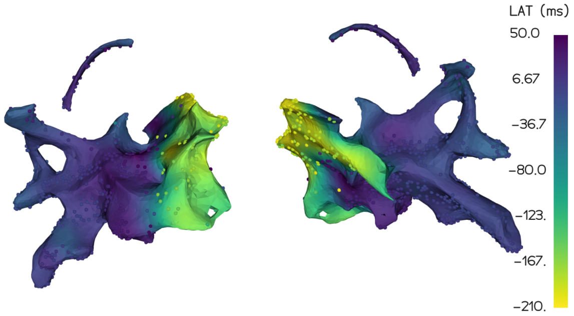

(b)

Figure 9: Patient D, Map 6 interpolation by MAGIC-LAT (a) and GPMI (b) for $m = 100$ input LAT observations. The interpolations color the manifold surface underneath the known LAT observations. MAGIC-LAT very closely approximates the sharp early-meets-late transition region, while GPMI erroneously smooths it out with a wide green to purple transition band.
